# Supplementary material for: Research on the speed fluctuation control of diesel engine under load changes via an improved sparrow algorithm
Source: PLoS One. 2025 Aug 1;20(8):e0329266. doi: 10.1371/journal.pone.0329266 (PMC12316253; doi:10.1371/journal.pone.0329266)
Supplement: Attachment — Submitted filename: My review.pdf [file pone.0329266.s004.pdf]

Concerns

1. In the following sentence [6] will be before full stop.
   Traditional PID controllers cannot perform online parameter tuning, and in complex systems such as diesel engines, which are nonlinear and time-varying, their control effects are often unsatisfactory. [6]
2. In the introduction section, performance of cited studies could be added briefly.
3. "ELM" is mentioned in the below paragraph but what it stands for is missing:
   Zhang [27] improved the sparrow algorithm for an ELM neural network to propose a method for the inversion of soil parameters for underground space development and the prediction of deformation of underground structures.
4. "RPM" is written multiple times but what it stands for is not described anywhere.
5. Reference of basic equations 1, 2, 4, 5, 11 should be added.
6. In the following equation d-phi is not explained:

$$\frac{dm}{d\varphi} = \frac{dm_s}{d\varphi} + \frac{dm_e}{d\varphi} + \frac{dm_b}{d\varphi}$$

7. In the description of following equation, some variables are not explained:

$$V_s = \frac{\Pi D^2}{4}\{\frac{S}{\varepsilon-1} + \frac{S}{2}[(1 + \frac{1}{\lambda}\sqrt{1 - \lambda^2 \sin^2\left(\frac{\Pi}{180}\varphi\right)}\ ]\} \quad (4)$$

8. Qw is not explained for the following equation

$$\frac{dQ_w}{d\varphi} = \sum_{i=1}^{3} \frac{dQ_{wi}}{d\varphi} = \frac{1}{w}\sum_{i=1}^{3} \alpha_g \cdot A_i(T - T_{wi})$$

9. a_g is written in paragraph, whereas the equation is

$$\alpha_g = 130 \cdot D^{0.2} \cdot p^{0.8} \cdot T^{-0.53} \left[C_1 \cdot C_m + C_2 \cdot \frac{V_s \cdot T_{cj}}{P_{cj} \cdot V_{cj}}(p - p_{c0})\right]^{0.8} (8)$$

10. Is it "Engine modle" in Fig. 3?
11. It should be explained clearly that why sparrow algorithm is specifically considered for optimizing the algorithm.
12. In eq. 14, ST is not explained. Second, the link between R2 with X is missing.
13. Derivation of eq. 16 lacks clarity.

14. The derivation of eq. 17 and 18 must be added. How the authors finally reached at these equations?
15. Fig. 9 is written twice with same title.
16. It would be interesting to see how the cylinder pressure behaves under different loads.
17. Since authors propose improved SSA, I strongly recommend that its flow chart should be added in the manuscript.
18. It should mentioned that how would the system behave under transient operating conditions?
