# Supplementary material for: Research on the speed fluctuation control of diesel engine under load changes via an improved sparrow algorithm
Source: PLoS One. 2025 Aug 1;20(8):e0329266. doi: 10.1371/journal.pone.0329266 (PMC12316253; doi:10.1371/journal.pone.0329266)
Supplement: Attachment — Submitted filename: Response to Reviewers.docx [file pone.0329266.s006.docx]

Dear Editor and Reviewers：

We sincerely thank the editor and all reviewers for their valuable feedback that we have used to improve the quality of our manuscript. The reviewer comments are laid out below in italicized font and specific concerns have been numbered. Our response is given in normal font and changes to the manuscript are given in the red text.

***Q1: Clarity in Problem Statement and Objectives:The introduction should provide a clearer and more concise statement of the research problem. While the paper discusses the limitations of traditional PID control, the connection to the improved sparrow algorithm should be articulated more explicitly. The motivation for selecting this specific algorithm needs stronger justification.***

A1: Thanks for your suggestion. In response to the above problems, we have made changes in the article as follows：Line 65 shows that the Sparrow algorithm has higher accuracy and faster response speed than other algorithms, and three related literature articles are added at the end of the paragraph to show that the algorithm is superior to other algorithms in the process of use. In line 73, the case literature is analyzed and summarized, and the algorithm improvement direction is proposed by considering how to combine the application of the algorithm with the speed control of diesel engine.

The specific changes are as follows：

In recent years, the sparse search algorithm (SSA), an emerging intelligent optimization algorithm[24], has performed well in a variety of optimization problems owing to its simplicity, efficiency, and good adaptability[25,26,27].

~~The above study shows that the SSA performs well for various engineering problems~~~~.~~

Above scholars have addressed the inherent defects of the pigeon-inspired algorithm, such as population initialization imbalance, slow convergence speed, and tendency to get trapped in local optima, by proposing different solutions. In the diesel engine speed control system, the optimization accuracy of control parameters directly affects the speed regulation performance, imposing stricter requirements on the global search capability and convergence accuracy of the optimization algorithm.

~~This study aims to propose an improved sparrow algorithm for the problem of diesel speed control under load variation. It is applied to the parameter optimization of a fuzzy PID controller, achieving stable control of the diesel engine speed under load changes by optimizing the proportional and quantization factors of the fuzzy PID.~~

Therefore, this study proposes an improved Squirrel Search algorithm to address the problem of the algorithm easily falling into local optima due to the high precision requirements for parameter adjustment in diesel engine speed control, and achieves stable control of the diesel engine speed under load changes by optimizing the proportion factor and quantization factor of the FPID.

***Q2:*** ***Experimental Validation and Benchmarking:The manuscript lacks a direct comparison of the proposed method with other state-of-the-art algorithms. It would be beneficial to include additional benchmark tests against other optimization techniques such as Genetic Algorithm (GA) or Particle Swarm Optimization (PSO) in practical diesel engine control scenarios.***

A2: We sincerely thank you for your valuable suggestions and believe they will be very helpful in improving the quality of the manuscripts. Therefore, we added the particle swarm optimization algorithm (PSO) to the comparative test and presented the experimental results in Figure 8.

***Q3: Algorithm Implementation Details:The section describing the improved sparrow algorithm lacks sufficient implementation details. The modifications to the standard sparrow search algorithm should be elaborated on with mathematical derivations or pseudo-code to enhance reproducibility.***

A3: Thanks for your suggestion. We had supplemented the algorithmic implementation process by adding the pseudo-code of the ISSA algorithm at line 287 in the manuscript to enhance the reproducibility of the algorithm.

The pseudo-code implementation process of improving the sparrow algorithm is as follows:

**Algorithm 1** The ISSA Algorithm

**Input：**

n: the number of sparrows

PD: the number of the producer sparrows

SD: the number of the sparrows which perceive danger

Iter_max_: the total number of iterations

R_2_: the alarm value

**output：**

**X_best_**: the global optimal position

**f_best_**: the optimal solution

1: Using (21) to Initialize a population of n sparrows

2:**whiel(t<Iter_max_)**

3: Calculating the fitness values of the individuals;

4: Ranking the fitness values and finding the current best individual and the current worst individual;

5: **For** I =1: PD

6: Using (20) to update the producer position;

7: end for

8: **For** I =(PD+1): n

9: Using (21) to update the scrounger position;

10: **end for**

11: **For** I = 1: SD

12: Using (22) to update the Scout Position;

13: **end for**

14: Obtaining the current new position;

15： **if** (the new position is better than the best position)

16: Replacing the best position with the new position

17: **end if**

18: t=t+1;

19: **end while**

20: return **X_best_**, **f_best_**;

***Q4: Statistical Analysis of Results:The performance improvement claims are primarily based on numerical results, but no statistical validation is presented. Confidence intervals, variance analysis, and standard deviations should be included to validate the consistency and robustness of the results.***

A4: Thank you for your valuable feedback, the issue you mentioned is very crucial. The improvement of algorithm performance needs to include Confidence intervals, variance analysis, and standard deviations. Therefore, in line 291 of the text and Table 2, it is pointed out that all tests were run independently 30 times to eliminate the influence of random numbers, with 1000 iterations and a population size of 100 for the algorithm. Reference “A novel inversion approach for seepage parameter of concrete face rockfill dams based on an enhanced sparrow search algorithm” is used for algorithm validation, using the optimal value, average value, and standard deviation as the basis for testing. And combine the three tables into one table

The specific changes are as follows：

~~To validate the performance and optimization results of the improved SSA, four benchmark functions with significant optimization features were selected.~~ ~~These functions were tested in 30 dimensions with a population size of 100, and 30 tests were performed to evaluate the improved algorithm, as listed in Table 2.~~

To verify the performance and optimization results of the improved SSA, four benchmark functions with significant optimization characteristics were selected. These functions were independently run 30 times to avoid the influence of random numbers, with 1000 iterations set and a population size of 50. he specific dimensions, ranges, and extreme values of the test functions are detailed in Table 2.

~~Table 3. Optimal values of test function search results~~

| ~~Function~~ | ~~ISSA~~ | ~~SSA~~ | ~~GWO~~ | ~~PSO~~ |
| --- | --- | --- | --- | --- |
| ~~F1~~ | ~~0~~ | ~~4.82E−98~~ | ~~5.86E-86~~ | ~~3.91E-3~~ |
| ~~F2~~ | ~~1.89E-22~~ | ~~5.44E-13~~ | ~~25.17~~ | ~~2.30~~ |
| ~~F3~~ | ~~0~~ | ~~0~~ | ~~0~~ | ~~3.97~~ |
| ~~F4~~ | ~~0~~ | ~~0~~ | ~~0.01~~ | ~~0.17~~ |

~~Table 4. Average values of the test function search results~~

| ~~Function~~ | ~~ISSA~~ | ~~SSA~~ | ~~GWO~~ | ~~PSO~~ |
| --- | --- | --- | --- | --- |
| ~~F1~~ | ~~0~~ | ~~2.55E-55~~ | ~~1.39e-85~~ | ~~0.21~~ |
| ~~F2~~ | ~~1.07E-07~~ | ~~1.79E-02~~ | ~~25.19~~ | ~~7.74~~ |
| ~~F3~~ | ~~0~~ | ~~1.02e-12~~ | ~~0~~ | ~~4.75~~ |
| ~~F4~~ | ~~0~~ | ~~1.66e-13~~ | ~~0.02~~ | ~~0.21~~ |

Table 3. Comparison of test results of different algorithms.

| Indicator | Function | ISSA | SSA | GWO | PSO |
| --- | --- | --- | --- | --- | --- |
| Beast value | F1 | 0 | 4.82E−98 | 5.86E-86 | 3.91E-3 |
|  | F2 | 3.31E-07 | 1.03E-05 | 5.42E-05 | 0.017 |
|  | F3 | 0 | 0 | 0 | 3.97 |
|  | F4 | 0 | 0 | 0.01 | 0.17 |
| Average values | F1 | 0 | 2.5E-52 | 8.07E-85 | 0.047 |
|  | F2 | 6.85E-06 | 4.16E-04 | 2.84E-04 | 0.104 |
|  | F3 | 0 | 1.51E-14 | 0 | 7.721 |
|  | F4 | 0 | 1.87E-16 | 0.023 | 0.154 |
| Strand deviation | F1 | 0 | 1.38E-51 | 1.66587E-84 | 0.057 |
|  | F2 | 6.14E-06 | 4.83E-04 | 1.57E-04 | 0.067 |
|  | F3 | 0 | 4.70E-14 | 0 | 1.919 |
|  | F4 | 0 | 1.87E-16 | 0.023 | 0.154 |

~~In this study, four optimization algorithms were compared, namely, particle swarm optimization (PSO), gray wolf optimization (GWO), the Sparrow algorithm (SSA), and the improved Sparrow algorithm (ISSA). Figure 4 and Table 3 show the convergence curves of these algorithms on the four test functions, from which it can be seen that the ISSA algorithm exhibits the best performance among all test functions. Table 4 shows the average values after 30 tests of the algorithm, demonstrating the high stability and accuracy of the ISSA algorithm compared with the four other algorithms. The results indicate that the ISSA algorithm exhibits stronger stability among the four test functions, suggesting that the algorithm can overcome local optimum issues and obtain the best test results.~~

In this study, four optimization algorithms were compared, namely, particle swarm optimization (PSO), gray wolf optimization (GWO), the Sparrow algorithm (SSA), and the improved Sparrow algorithm (ISSA). Figure 4 show the convergence curves of these algorithms on the four test functions, from which it can be seen that the ISSA algorithm exhibits the best performance among all test functions. Table 3 shows the optimal values, average values, and standard deviations of four algorithms after 30 independent runs. It can be found that the ISSA algorithm performs the best performance under three indices, and it can find the most extreme values in the F1, F3, F4 test functions, and is closer to the extreme value than the other algorithms in the F2 test function. At the same time, both the average values and standard deviations after 30 runs are better than those of the other algorithms.

***Q5: Discussion on Computational Complexity:The manuscript does not sufficiently address the computational complexity of the improved algorithm compared to standard SSA. The trade-off between computational cost and performance improvement should be discussed in more detail, especially for real-time applications.***

A5: Thanks for your valuable comments on improving the computational complexity of the ISSA and its impact on real-time applications. Here is my reply to this question.

In the original algorithm, the computational complexity involves the roles of explorers, followers, and sentinels, fitness evaluation, sorting, and selection decisions. The main improvements in the ISSA algorithm include circular chaotic mapping initialization, dynamic search factor, sine-cosine strategy, and Lévy flight strategy. These improvements do not add more operation steps or structures, so overall, the ISSA maintains the same asymptotic complexity as SSA, but the ISSA algorithm has faster convergence speed, which can be demonstrated in Figure 8. For real-time applications like diesel engines, the algorithm is mainly used for offline optimization and online fine-tuning of fuzzy rules.

***Q6:Grammar and Syntax Corrections: There are several grammatical errors throughout the text. For example, "However, the improved algorithm improves the control effect but also increases the computational complexity" should be rephrased to "However, while the improved algorithm enhances the control effect, it also increases computer***

A6: Thanks for your valuable comments. We carefully check and revise the errors you raised, and at the same time carefully check the article to correct the grammatical errors in the text, and attach a test report.

~~However, the improved algorithm improves the control effect but also increases the computational complexity~~

However, while the improved algorithm enhance the control effect, it also increase the compute

***Q7: Consistency in Terminology: The terms "fuzzy PID control" and "FPID" should be used consistently throughout the manuscript to avoid confusion.***

A7: Thanks for your valuable feedback. This is an imprecise error which I have corrected. Fuzzy PID is replaced by FPID.

***Q8: Figure and Table Captions: Some figure captions lack detailed descriptions. For example, Figure 9 should explicitly state what the plotted data represent.***

A8: Thanks for your valuable feedback. In my manuscript, Figure 9 appeared twice, and this error has been corrected. Figure 9 represents the response curve of the diesel engine from idle to the target speed of 2000 RPM. Figure 10 represents the random load under load changes.

具体修改如下：~~Figure 10. Load Variation~~ Figure 10.different load 6-10$N\cdot M$

***Q9: Units and Notations: Ensure consistency in units (e.g., "RPM" vs. "r/min"). Some values are given in different formats across sections.***

A9: Thanks for your valuable feedback. I have unified the units and replaced them with RPM.

***Q10: Reference Formatting: Some citations are not formatted according to PLOS ONE guidelines. Ensure uniformity in references, particularly in citing author names and publication years.***

A10: Thank you for your valuable comments. I have carefully revised the references according to the requirements of your journal, as follows:

~~[1]Sujesh G ,Ramesh S .Modeling and control of diesel engines: A systematic review[J].Alexandria Engineering Journal,2018,57(4):4033-4048.~~

~~[2]Yeom J ,Jung S ,Yoon J .An experimental study on the application of oxygenated fuel to diesel engines[J].Fuel,2019,248262-277.~~

~~[3]Frank L H I ,Bolan L ,Fanshuo L , et al.Review of Artificial Intelligent Algorithms for Engine Performance, Control, and Diagnosis[J].Energies,2023,16(3):1206-1206.~~

~~[4]Åström K J, Hägglund T. PID control[J]. IEEE Control Systems Magazine, 2006, 1066.~~

~~[5]Borase R P, Maghade D K, Sondkar S Y, et al. A review of PID control, tuning methods and applications[J]. International Journal of Dynamics and Control, 2021, 9: 818-827.~~

~~[6]Somefun O A, Akingbade K, Dahunsi F. The dilemma of PID tuning[J]. Annual Reviews in Control, 2021, 52: 65-74.~~

~~[7]Padula F, Visioli A. Tuning rules for optimal PID and fractional-order PID controllers[J]. Journal of process control, 2011, 21(1): 69-81.~~

~~[8]Zhao C, Guo L. PID controller design for second order nonlinear uncertain systems[J]. Science China Information Sciences, 2017, 60: 1-13.~~

~~[9]Yu W, Rosen J. Neural PID control of robot manipulators with application to an upper limb exoskeleton[J]. IEEE Transactions on cybernetics, 2013, 43(2): 673-684.~~

~~[10]Xu J, Xiong Z, Bhattacharyya S P. PIDNet: A real-time semantic segmentation network inspired by PID controllers[C]. Proceedings of the IEEE/CVF conference on computer vision and pattern recognition. 2023: 19529-19539.~~

~~[11]Sahib M A, Ahmed B S. A new multiobjective performance criterion used in PID tuning optimization algorithms[J]. Journal of advanced research, 2016, 7(1): 125-134.~~

~~[12]Mishra P, Kumar V, Rana K P S. A fractional order fuzzy PID controller for binary distillation column control[J]. Expert Systems with Applications, 2015, 42(22): 8533-8549.~~

~~[13]Najariyan M, Zhao Y. Granular fuzzy PID controller[J]. Expert Systems with Applications, 2021, 167: 114182.~~

~~[14]Cong S, Liang Y. PID-like neural network nonlinear adaptive control for uncertain multivariable motion control systems[J]. IEEE Transactions on Industrial Electronics, 2009, 56(10): 3872-3879.3~~

~~[15]Zhao Y, Du X, Xia G, et al. A novel algorithm for wavelet neural networks with application to enhanced PID controller design[J]. Neurocomputing, 2015, 158: 257-267.~~

~~[16]Zeng B ,Shen Q ,Wang G , et al. Research on Diesel Engine Speed Control Based on Improved Salp Algorithm[J].Processes,2023,11(11):~~

~~[17]~~~~Zhu Cunxi.~~ ~~Research on Speed Control Strategy of Marine Medium - speed Engine Based on Genetic Algorithm [D]. Harbin Engineering University, 2023.~~

~~[18]Zhuo H ,Weihao G ,Kege Z , et al.Optimization of PID control parameters for marine dual-fuel engine using improved particle swarm algorithm[J].Scientific Reports,2024,14(1):12681-12681.~~

~~[19]Ding S L, He S F, Tu B Q, et al.~~ ~~Model‐Based Control with Active Disturbance Rejection Algorithm for a Diesel Engine[J]. Complexity, 2023.~~

~~[20]Xiang D ,Ying H ,Yanwu G , et al.~~ ~~Fuzzy-PID Speed Control of Diesel Engine Based on Load Estimation [J]. SAE International Journal of Engines, 2015, 8 (4): 1669-1677.~~

~~[21]Jie Y ,Fu Y S ,Bo-Chiuan C , et al.~~~~Application of Adaptive Idle Speed Control on V2 Engine[J].SAE International Journal of Engines,2015,9(1):458-465.~~

~~[22]Zhangmiaoge L ,Zhouxiao L ,Jianzhao L , et al.~~ ~~Thermal management with fast temperature convergence based on optimized fuzzy PID algorithm for electric vehicle battery [J]. Applied Energy, 2023, 352~~

~~[23]Azizi M ,Ejlali G R ,Ghasemi M A S , et al.~~~~Upgraded Whale Optimization Algorithm for fuzzy logic based vibration control of nonlinear steel structure[J].Engineering Structures,2019,19253-70.~~

~~[24]Xue J, Shen B.~~ ~~A novel swarm intelligence optimization approach: sparrow search algorithm[J]. Systems science & control engineering, 2020, 8(1): 22-34.~~

~~[25]Li W ,Zhu H ,Yang F , et al.~~~~Storm-time ionospheric model over Yunnan-Sichuan area of China based on the SSA-ConvLSTM-BiLSTM algorithm[J].GPS Solutions,2025,29(2):77-77.~~

~~[26]Yuan P ,Zhao W ,Zhao Y .~~~~Intelligent prediction algorithm of ship roll and pitch motion based on SSA- optimized BiLSTM network[J].Ocean Engineering,2025,320120331-120331.~~

~~[27]Du X ,Chen K ,Du H , et al.~~~~TS-SSA: An improved two-stage sparrow search algorithm for large-scale many-objective optimization problems.[J].PloS one,2025,20(3):e0314584.~~

~~[28]Zhang Zhihe, Zhou Kaihong, Zhu Mengyan. Control of Lower Limb Rehabilitation Robot Based on Improved SSA - Optimized PID[J]. Modern Electronics Technique, 2024, 47(13): 153 - 159.~~

~~[29]Liang Jianguo, Huang Haozhong, Long Huigui, et al. Research on Turbojet Engine Control Based on Sparrow Search Algorithm[J]. Journal of Guangxi University (Natural Science Edition), 2024, 49(03): 553 - 562.~~

~~[30]Zhang Kunyong, Su Zhengkai, Nie Meijun, et al. Soil Parameter Inversion and Deformation Prediction Method Based on ISSA - ELM[J]. Journal of Basic Science and Engineering, 2024, 32(05): 1434 - 1448.~~

~~[31]Xue Hai, Ye Cenglin, He Yongfeng, et al. Adaptive Determination Method of Stress Spectrum Threshold for Metro Frame Based on ISSA - VMD[J]. China Railway Science, 2024, 45(04): 180 - 188.~~

~~[32]Isermann R.~~ ~~Engine modeling and control[J]. Berlin: Springers Berlin Heidelberg, 2014, 1017.~~

~~[33]Sahu K R ,Panda S ,Yegireddy K N .~~~~A novel hybrid DEPS optimized fuzzy PI/PID controller for load frequency control of multi-area interconnected power systems[J].Journal of Process Control,2014,24(10):1596-1608.~~

~~[34]Rui Y ,Yongbao L ,Youhong Y , et al.~~~~Hybrid improved particle swarm optimization-cuckoo search optimized fuzzy PID controller for micro gas turbine[J].Energy Reports,2021,75446-5454.~~

[1] Sujesh G, Ramesh S. Modeling and control of diesel engines: A systematic review. Alexandria Engineering Journal. 2018;57(4):4033-48.
[2] Yeom J-K, Jung S-H, Yoon J-H. An experimental study on the application of oxygenated fuel to diesel engines. Fuel. 2019;248:262-77.
[3] Frank IHL, Bolan L, Fanshuo L, Junwei Z, Ben L, Peng W. Review of Artificial Intelligent Algorithms for Engine Performance, Control, and Diagnosis. Energies. 2023;16(3):1206-.
[4] Åström KJ, Hägglund T. PID control. IEEE Control Systems Magazine. 2006;1066:30-1.
[5] Borase RP, Maghade D, Sondkar S, Pawar S. A review of PID control, tuning methods and applications. International Journal of Dynamics and Control. 2021;9:818-27.
[6] Somefun OA, Akingbade K, Dahunsi F. The dilemma of PID tuning. Annual Reviews in Control. 2021;52:65-74.
[7] Padula F, Visioli A. Tuning rules for optimal PID and fractional-order PID controllers. Journal of process control. 2011;21(1):69-81.
[8] Zhao C, Guo L. PID controller design for second order nonlinear uncertain systems. Science China Information Sciences. 2017;60:1-13.
[9] Yu W, Rosen J. Neural PID control of robot manipulators with application to an upper limb exoskeleton. IEEE Transactions on cybernetics. 2013;43(2):673-84.
[10] Xu J, Xiong Z, Bhattacharyya SP, editors. PIDNet: A real-time semantic segmentation network inspired by PID controllers. Proceedings of the IEEE/CVF conference on computer vision and pattern recognition; 2023.
[11] Sahib MA, Ahmed BS. A new multiobjective performance criterion used in PID tuning optimization algorithms. Journal of advanced research. 2016;7(1):125-34.
[12] Mishra P, Kumar V, Rana K. A fractional order fuzzy PID controller for binary distillation column control. Expert Systems with Applications. 2015;42(22):8533-49.
[13] Najariyan M, Zhao Y. Granular fuzzy PID controller. Expert Systems with Applications. 2021;167:114182.
[14] Cong S, Liang Y. PID-like neural network nonlinear adaptive control for uncertain multivariable motion control systems. IEEE Transactions on Industrial Electronics. 2009;56(10):3872-9.
[15] Zhao Y, Du X, Xia G, Wu L. A novel algorithm for wavelet neural networks with application to enhanced PID controller design. Neurocomputing. 2015;158:257-67.
[16] Zeng B, Shen Q, Wang G, Wang Y, Zhao Y, He S, Yu X. Research on Diesel Engine Speed Control Based on Improved Salp Algorithm. Processes. 2023;11(11):3092.
[17] Zhu Cunxi. Research on Speed Control Strategy of Marine Medium - speed Engine Based on Genetic Algorithm [Master]2023.
[18] Hu Z, Guo W, Zhou K, Wang L, Wang F, Yuan J. Optimization of PID control parameters for marine dual-fuel engine using improved particle swarm algorithm. Scientific Reports. 2024;14(1):12681.
[19] Ding S-L, He S-F, Tu B-Q, Liu J-J, Wang Y-Y, Song E-Z. Model‐Based Control with Active Disturbance Rejection Algorithm for a Diesel Engine. Complexity. 2023;2023(1):8429922.
[20] Di X, Huang Y, Ge Y, Li G, Hu M. Fuzzy-PID speed control of diesel engine based on load estimation. SAE International Journal of Engines. 2015;8(4):1669-77.
[21] Yin J, Syu YF, Chen B-C, Wu Y-Y, Liang YC, Tsai H-C. Application of adaptive idle speed control on V2 engine. SAE International Journal of Engines. 2016;9(1):458-65.
[22] Liu Z, Liu Z, Liu J, Wang N. Thermal management with fast temperature convergence based on optimized fuzzy PID algorithm for electric vehicle battery. Applied Energy. 2023;352:121936.
[23] Azizi M, Ejlali RG, Ghasemi SAM, Talatahari S. Upgraded Whale Optimization Algorithm for fuzzy logic based vibration control of nonlinear steel structure. Engineering Structures. 2019;192:53-70.
[24] Xue J, Shen B. A novel swarm intelligence optimization approach: sparrow search algorithm. Systems science & control engineering. 2020;8(1):22-34.
[25] Li W, Zhu H, Yang F, Wen C, Shi S, Zhao D, et al. Storm-time ionospheric model over Yunnan-Sichuan area of China based on the SSA-ConvLSTM-BiLSTM algorithm. GPS Solutions. 2025;29(2):77.
[26] Yuan P, Zhao W, Zhao Y. Intelligent prediction algorithm of ship roll and pitch motion based on SSA- optimized BiLSTM network. Ocean Engineering. 2025;320:120331-.
[27] Du X, Chen K, Du H, Qiao Z. TS-SSA: An improved two-stage sparrow search algorithm for large-scale many-objective optimization problems. PloS one. 2025;20(3):e0314584.
[28] Zhang Zhihe, Zhou Kaihong, Zhu Mengyan. Control of Lower Limb Rehabilitation Robot Based on Improved SSA - Optimized PID. Modern Electronics Technique, 2024, 47(13): 153 - 159.
[29] Liang Jianguo, Huang Haozhong, Long Huigui, et al. Research on Turbojet Engine Control Based on Sparrow Search Algorithm. Journal of Guangxi University (Natural Science Edition), 2024, 49(03): 553 - 562.
[30] Zhang Kunyong, Su Zhengkai, Nie Meijun, et al. Soil Parameter Inversion and Deformation Prediction Method Based on ISSA - ELM. Journal of Basic Science and Engineering, 2024, 32(05): 1434 - 1448.
[31] Xue Hai, Ye Cenglin, He Yongfeng, et al. Adaptive Determination Method of Stress Spectrum Threshold for Metro Frame Based on ISSA - VMD. China Railway Science, 2024, 45(04): 180 - 188.
[32] Isermann R. Engine modeling and control. Berlin: Springers Berlin Heidelberg, 2014, 1017.
[33] Sahu RK, Panda S, Yegireddy NK. A novel hybrid DEPS optimized fuzzy PI/PID controller for load frequency control of multi-area interconnected power systems. Journal of Process Control. 2014;24(10):1596-608.
[34] Rui Y, Yongbao L, Youhong Y, Xing H, Hongsong L. Hybrid improved particle swarm optimization-cuckoo search optimized fuzzy PID controller for micro gas turbine. Energy Reports. 2021;7:5446-54.

***Q11:*** ***Clarification on Experimental Conditions: The manuscript does not specify whether real-world diesel engine tests were conducted or if all results are from simulations. This should be explicitly stated in the methodology.***

A11: Thank you for your valuable comments, this question is very necessary, so we explain in this article that the results of Chapter 5 are obtained by simulation calculations, and an accurate diesel engine model is obtained by comparing it with the experimental bench in Chapter 2, and the algorithm is brought into this model and declare that the results are from the simulation.

***Q12: Equation Formatting and Explanation: Some equations lack proper explanation, such as Equation (4). The meaning of variables should be explicitly defined.***

A12: Thank you for your valuable comments. After careful review, we found that there is a lack of specific explanations regarding D and φ. We have explained these parameters：

$V_{s}=\frac{\Pi D^{2}}{4}\{\frac{S}{\varepsilon-1}+\frac{S}{2}[(1+\frac{1}{\lambda}\sqrt{1-\lambda^{2}\sin^{2}（\frac{\Pi}{180}\varphi）}]\}$	(4)

where $V_{S}$ is the working volume of the cylinder, $L$;D is the cylinder diameter; $\lambda$ is the connecting rod-to-crank ratio; $s$ is the cylinder stroke; $\varepsilon$ is the compression ratio and φ is the ccrankshaft angle.

***Q13: Abstract Refinement: The abstract should be more concise and should highlight the key contributions in a clearer manner, including the percentage improvements achieved.***

A13: Thank you for your valuable comments. We have modified the issue you pointed out, replacing the previous specific data modification with a percentage increase as follows: The duration for speed fluctuation stabilization under load changes of 8 and 10 is diminished by ~~0.3 s~~ 18%and ~~0.7 s~~30%, respectively, and the fluctuation deviation of the speed is reduced by ~~10 RPM~~7% and ~~20 RPM~~12%, respectively.

***Q14:*** ***Improved Transition Between Sections: Some sections transition abruptly. For example, the shift from theoretical modeling to algorithm description should be smoother, with better contextual bridging.***

A14: Thank you for your valuable comments. We have made some improvements to the chapter structure, as follows：

~~Diesel engines are complex nonlinear systems, and in the face of environmental changes, changes in operating conditions, and other disturbance factors, their operating characteristics often change. Fuzzy logic can flexibly adjust the parameters of a PID controller according to the operating state of the system, thus enhancing the adaptability of the controller to complex and uncertain systems[33]. Therefore, this study utilized a combination of fuzzy logic and a PID controller to design a suitable controller for a diesel engine speed control system.~~

Through model verification, it is proved that the dynamic characteristics of the diesel engine are highly consistent with the simulation results. On this basis, this section will propose a fuzzy PID control method based on an improved sparrow search algorithm to address the limitations of traditional PID control in nonlinear systems[33].

***Q15: Addressing Limitations and Future Work: The paper should include a more detailed discussion on the limitations of the proposed method and potential areas for future research, such as real-time implementation challenges or hybrid optimization strategies.***

A15: Thank you for your valuable comments. Regarding the issue of limitations and future work, we look forward to improvements in algorithm efficiency, the design of hybrid control strategies, and practical application verification, and expect further expansiona and briefly pointed out in the article.

~~Future research will further optimize the structure of the controller to improve its computational efficiency and practicality.~~

In the future, further research can be carried out on three aspects: algorithm efficiency improvement, hybrid control strategy design, and practical application verification

***Q16:In the following sentence*** ***[6] will be before full stop.Traditional PID controllers cannot perform online parameter tuning, and in complex systems such as diesel engines, which are nonlinear and time-varying, their control effects are often unsatisfactory.***

A16: Thank you for your valuable comments. The incorrect [6] locations have been corrected.

***Q17: In the introduction section, performance of cited studies could be added briefly.***

A17: Thank you for your valuable comments. The issue you mentioned has been modified as follows:

(1) Boshun Zeng [16]proposed a speed control method that combines the improved Salp algorithm with a PID controller to optimize the PID controller parameters of the diesel engine model through the improved Salp algorithm, aiming to improve the control accuracy and stability,during sudden changes in speed and load, the overshoot decreased by an average of more than 30.3% and more than 8.6%, respectively.
(2) Hu [18]improved the Particle Swarm Optimization(PSO) algorithm and applied it to dual-fuel engine control to enhance speed control stability,The response time reduced by 0.47 s and the maximum overshoot reduced by 98.43%.
(3) Di et al.[20] combined PID control with fuzzy control and proposed a parameter self-tuning fuzzy PID controller for diesel engine speed control,which reduced the speed change rate from 26.61% to 7.51%, and the adjustment duration of fuel injection from 4.91s to 2.07s, which effectively improved the stability of the system.
(4) Jie [21]et al. applied adaptive control to a V2 engine and achieved better performance than traditional PID control, and the results showed that the feedforward adaptive controller could significantly improve the tracking time by about 40% under ramp loads.
(5) Liu [22]proposed an optimized fuzzy PID algorithm applied to a fast temperature control system for automotive batteries, and the results showed that the comprehensive performance of the fuzzy PID composite controller was better than that of the ordinary PID controller and basic fuzzy controller,the temperature control time is reduced by approximately 76%.
(6) Zhang Zhihuo[28]improved the SSA and applied it to the control of a lower-limb rehabilitation robot, and the results showed that the ISSA-PID control significantly reduced the error of the hip and knee joints by 63.3% and 72.5%, respectively, and achieved accurate gait tracking.
(7) Liang [29] addressed the problem of parameter optimization of the traditional PID controller and proposed a control method based on the sparse search algorithm (SSA). Compared with the traditional PID control method, the response speed of the proposed method is increased by 64.1% and the interference adjustment time is shortened by 52.3%.
(8) Zhang [30]improved the sparrow algorithm for an Extreme Learning Machine (ELM) neural network to propose a method for the inversion of soil parameters for underground space development and the prediction of deformation of underground structures.The relative error of the horizontal displacement monitoring value of the ground wall is reduced by 10%.
(9) Xue [31] optimized the variational modal decomposition process of the improved sparrow search algorithm and the total number of stress rainflow cycles was reduced by 17.1%, and the actual damage was reduced by 7.8% compared with the results obtained by the traditional method, which effectively reflected the fatigue effect caused by stress and improved the efficiency of stress spectrum compilation.

***Q18:“ELM” is mentioned in the below paragraph but what it stands for is missing:***

***Zhang [27] improved the sparrow algorithm for an ELM neural network to propose a method for the inversion of soil parameters for underground space development and the prediction of deformation of underground structures. “******ELM”***

A18: Thank you for your valuable comments. The full form of ELM is Extreme Learning Machine, and we have already added it in the article.

***Q19: “RPM” is written multiple times but what it stands for is not described anywhere.***

A19: Thank you for your valuable comments. Explanation of RPM was mentioned in the abstract, in line 21 of the article.

***Q20: Reference of basic equations 1, 2, 4, 5, 11 should be added.***

A20: Thank you for your valuable comments. Your advice is accurate, but we have already indicated in line 97 that the following formula references are from [32]Isermann R. Engine modeling and control. Berlin: Springers Berlin Heidelberg, 2014, 1017.

***Q21:*** ***In the following equation d-phi is not explained:***

A21: Thank you for your valuable comments. We have made the modifications. In the equation,

$\frac{dm}{d\varphi}=\frac{{dm}_{s}}{d\varphi}+\frac{{dm}_{e}}{d\varphi}+\frac{{dm}_{b}}{d\varphi}$	(1)

$d\varphi$ is the differential of the crankshaft angle

***Q22:*** ***In the description of following equation, some variables are not explained***

A22: Thank you for your valuable comments. We have made the modifications.

$V_{s}=\frac{\Pi D^{2}}{4}\{\frac{S}{\varepsilon-1}+\frac{S}{2}[(1+\frac{1}{\lambda}\sqrt{1-\lambda^{2}\sin^{2}（\frac{\Pi}{180}\varphi）}]\}$	(4)

where $V_{S}$ is the working volume of the cylinder, $L$;D is the cylinder diameter; $\lambda$ is the connecting rod-to-crank ratio; $s$ is the cylinder stroke; $\varepsilon$ is the compression ratio and φ is the ccrankshaft angle.

***Q23:*** ***Qw is not explained for the following***

A23: Thank you for your valuable comments. We have made the modifications.

$\frac{dQ_{w}}{d\varphi}=\sum_{i=1}^{3} \frac{dQ_{wi}}{d\varphi}=\frac{1}{w}\sum_{i=1}^{3} \alpha_{g}\cdot A_{i}\left( T-T_{wi} \right)$	(7)

$Q_{w}$ *isis the heat transfer from the cylinder wall under the angle of the unit crankshaft,* $J/^{\circ}CA$

***Q24:*** ***a_g is written in paragraph, whereas the equation is a_g***

A24: Thank you for your valuable comments. We have made the modifications. The changes are as follows:

The instantaneous average heat transfer coefficient $\alpha_{g}$ is expressed via the Woschni formula:

***Q25:*** ***Is it “Engine modle” in Fig. 3?***

A25: Thank you for your valuable comments. Figure 3 is a logical picture of the control principle, so it is represented in text. The diesel engine model is described in Table 1, where the diesel engine model is 168F.

***Q26:It should be explained clearly that why sparrow algorithm is specifically considered for optimizing the algorithm.***

A26: Thanks for your suggestion. In response to the above problems, we have made changes in the article as follows：Line 65 shows that the Sparrow algorithm has higher accuracy and faster response speed than other algorithms, and three related literature articles are added at the end of the paragraph to show that the algorithm is superior to other algorithms in the process of use. In line 73, the case literature is analyzed and summarized, and the algorithm improvement direction is proposed by considering how to combine the application of the algorithm with the speed control of diesel engine.

The specific changes are as follows：

In recent years, the sparse search algorithm (SSA), an emerging intelligent optimization algorithm[24], has performed well in a variety of optimization problems owing to its simplicity, efficiency, and good adaptability[25,26,27].

~~The above study shows that the SSA performs well for various engineering problems.~~

Above scholars have addressed the inherent defects of the pigeon-inspired algorithm, such as population initialization imbalance, slow convergence speed, and tendency to get trapped in local optima, by proposing different solutions. In the diesel engine speed control system, the optimization accuracy of control parameters directly affects the speed regulation performance, imposing stricter requirements on the global search capability and convergence accuracy of the optimization algorithm.

~~This study aims to propose an improved sparrow algorithm for the problem of diesel speed control under load variation. It is applied to the parameter optimization of a fuzzy PID controller, achieving stable control of the diesel engine speed under load changes by optimizing the proportional and quantization factors of the fuzzy PID.~~

Therefore, this study proposes an improved Squirrel Search algorithm to address the problem of the algorithm easily falling into local optima due to the high precision requirements for parameter adjustment in diesel engine speed control, and achieves stable control of the diesel engine speed under load changes by optimizing the proportion factor and quantization factor of the FPID.

***Q27: In eq. 14, ST is not explained. Second,*** ***the link between R2 with X is missing.***

A27: A24: Thank you for your valuable comments. We have made the modifications. The changes are as follows:

$X_{i,j}^{t+1}=\left\{ \begin{matrix} X_{i,j}^{t}\cdot\exp(-\frac{1}{\alpha\cdot iter_{max}} \\ X_{i,j}^{t}+Q\cdot L \end{matrix}\{ \begin{matrix} R_{2}<ST \\ R_{2}\geq ST \end{matrix} \right.$	(14)

where $t$ represents the number of iterations; $X_{i,j}^{t+1}$ indicates the value of the the row and j dimension at iteration t, $iter_{max}$ indicates the maximum number of iterations, $Q$ represents a random number that obeys a normal distribution, and $L$ is represented as a matrix of $1\times d$, where each element is 1. $R_{2}$ and $ST$ are safety values and warning values, respectively. When $R_{2}<ST$, sparrows perform a global search foraging; When $R_{2}\geq ST$, sparrows make random walks in a normal distribution.

***Q28:*** ***Derivation of eq. 16 lacks clarity.***

A28: Thank you for your valuable comments. eq. 16 is the original formula in cited literature [24] Xue J, Shen B. A novel swarm intelligence optimization approach: sparrow search algorithm. Systems science & control engineering. 2020;8(1):22-34.

***Q29:*** ***The derivation of eq. 17 and 18 must be added. How the authors finally reached at these equations?***

A29: Thank you for your valuable comments. eq. 17 is referenced from “[1] Altay V E, Alatas B. Bird swarm algorithms with chaotic mapping. Artificial Intelligence Review, 2020, 53(2): 1373-1414.” Equation 18 proves that Improved circle chaotic mapping is better than circle chaotic mapping by adjusting the parameters of Equation 17 and Figure 6.

***Q30:*** ***Fig. 9 is written twice with same title.Fig. 9***

A30: Thanks for your valuable feedback. In my manuscript, Figure 9 appeared twice, and this error has been corrected. Due to the addition of other pictures in front of the article, the serial number has changed, and the original figure 10 has been changed to figure 11

~~Figure 10. Load Variation~~ Figure 11.different load 6-10$N\cdot M$

***Q31:*** ***It would be interesting to see how the*** ***cylinder pressure behaves under different loads.***

A31: Thanks for your valuable feedback. It is very necessary to observe the cylinder pressure under different loads, but it is difficult to compare the cylinder pressures of different control algorithms under different loads in the comparative experiment of control algorithms. Therefore, in the second chapter of the model validation stage, cylinder pressures under different loads are added to verify the accuracy of the model. The specific modifications are as follows：

**Figure 3.** Cylinder pressure under different loads

***Q32: Since authors propose improved SSA, I strongly recommend that its flow chart should be added in the manuscript.***

A32: Thanks for your suggestion. Your opinion is very accurate. To improve the reproducibility of the code, I have chosen to display the pseudocode to demonstrate the reliability of the code in the paper

The pseudo-code implementation process of improving the sparrow algorithm is as follows:

**Algorithm 1** The ISSA Algorithm

**Input：**

n: the number of sparrows

PD: the number of the producer sparrows

SD: the number of the sparrows which perceive danger

Iter_max_: the total number of iterations

R_2_: the alarm value

**output：**

**X_best_**: the global optimal position

**f_best_**: the optimal solution

1: Using (21) to Initialize a population of n sparrows

2:**whiel(t<Iter_max_)**

3: Calculating the fitness values of the individuals;

4: Ranking the fitness values and finding the current best individual and the current worst individual;

5: **For** I =1: PD

6: Using (20) to update the producer position;

7: end for

8: **For** I =(PD+1): n

9: Using (21) to update the scrounger position;

10: **end for**

11: **For** I = 1: SD

12: Using (22) to update the Scout Position;

13: **end for**

14: Obtaining the current new position;

15： **if** (the new position is better than the best position)

16: Replacing the best position with the new position

17: **end if**

18: t=t+1;

19: **end while**

20: return **X_best_**, **f_best_**;

***Q33: It should mentioned that how would the system behave under transient operating conditions?***

A33: Thanks for your suggestion. System performance under transient conditions is a highly valuable research direction. Figure 12 can reflect to some extent the transient conditions of the diesel engine under load changes, and we will delve deeper into this through the establishment of a dynamic condition experimental platform in subsequent research
